# Antimicrobial photodynamic therapy for the treatment of angular cheilitis in critically Ill patients: a prospective study

**DOI:** 10.1007/s10103-026-04931-3

**Published:** 2026-06-29

**Authors:** André Luis Alves Borges, Sarah Emmily Melo da Silva, Tatiana Bernardo Farias Pereira, Jéssika Gulherme de Almeida Gonçalves, Éricka Janine Dantas da Silveira

**Affiliations:** Department of Oral Pathology, Federal University of Rio Grande do Norte, Natal, Brazil

**Keywords:** Photochemotherapy, Cheilitis, Intensive Care Units, Candidiasis, Oral, Low-Level Light Therapy

## Abstract

This preliminary prospective study aimed to evaluate the effectiveness of antimicrobial photodynamic therapy (aPDT) as an adjunct treatment for angular cheilitis (AC) in critically ill patients and to investigate its feasibility in a high-complexity hospital setting. The study included 13 patients admitted to the intensive care unit (ICU) of a referral infectious disease hospital in Northeastern Brazil. The aPDT protocol consisted of 0.1% methylene blue (10-minute pre-irradiation) followed by low-level laser therapy (660 nm, 4 J/point, 40 s) applied to three points per affected commissure. Clinical evolution was monitored at T1 (72 h), T2 (7 days), and T3 (follow-up), with lesions classified according to severity. Most patients presented severe systemic compromise, predominantly related to HIV infection and diabetes mellitus. In addition, 76.9% of patients exhibited concomitant oral candidiasis, and 92.3% were receiving systemic antifungal therapy. Complete lesion regression was observed in 40% of patients within 72 h (T1). By the final follow-up (T3), the overall clinical cure rate reached 61.5% (8/13), including cases of delayed resolution confirmed up to day 15. Although a 30.8% loss to follow-up occurred due to the inherent challenges of the intensive care setting, no adverse effects or complications were reported. Within the limitations of this preliminary study, aPDT demonstrated promising clinical applicability as an adjunctive approach for managing angular cheilitis in critically ill patients.

## Introduction

Although intensive care units (ICUs) rely on multidisciplinary teams to continuously monitor critically ill patients, the clinical characteristics and care conditions inherent to these patients may predispose them to the development of significant oral alterations. Factors such as the use of multiple medications, individual systemic conditions, orotracheal intubation, systemic infections, trauma, and inadequate oral hygiene have been identified as contributors to the development of oral complications in this population. These alterations may affect different oral tissues, including the mucosa, bone, teeth, and salivary glands [1], with opportunistic infections being among the most frequently reported conditions.

Among these infections, Candida species are recognized as the leading cause of fungal infections in hospitalized patients [2]. These microorganisms act as opportunistic pathogens capable of shifting from a commensal to a pathogenic state, a process facilitated by biofilm formation and systemic compromise, which may result in candidemia or disseminated infection affecting multiple organs [3, 4]. In the mouth, Candida infection can lead to oral candidiasis, which presents with various clinical manifestations. Acute and chronic forms may occur, including pseudomembranous candidiasis, the most characteristic presentation; erythematous candidiasis, the most frequent form and often associated with denture wearers; atrophic candidiasis, commonly affecting the tongue; and hyperplastic candidiasis, which has been associated with malignant transformation in approximately 12% of cases. An associated clinical manifestation of this infection is angular cheilitis (AC) [5, 6].

Care protocols in intensive care settings are based on therapeutic strategies aimed at reducing complications resulting from colonization by these microorganisms [3]. Treatment remains challenging; however, antifungal therapy is still considered the gold standard [5]. Additional fundamental principles must also be considered, particularly effective control of the infection source, as antifungal therapy alone is often insufficient, especially in the presence of biofilm-associated colonization [4, 7, 8].

In this context, antimicrobial photodynamic therapy (aPDT) has emerged as a promising alternative to conventional treatments, particularly in light of antifungal resistance, the emergence of multidrug-resistant species, and the consequent need for innovative strategies for the control of oral candidiasis (OC) [7, 8]. The effectiveness of this therapy depends on the choice of photosensitizers, with methylene blue (MB) standing out as a low-cost basic dye with well-established antimicrobial activity and rapid onset of action [8].

In this regard, aPDT has gained attention due to its well-established biological effects, low toxicity, and non-invasive nature [7, 8]. Therefore, this study aims to report a case series of patients undergoing aPDT in an ICU setting, with or without the use of antifungal therapy, focusing on evaluating its effectiveness either as a standalone treatment or in combination with systemic or topical antifungal agents, given the need to identify effective therapeutic approaches that do not further compromise the health of these patients.

## Methods

### Study desing and ethics

This study is a preliminary prospective observational investigation of the use of antimicrobial photodynamic therapy (aPDT) in the management of AC in patients admitted to a referral center for infectious diseases. The study was approved by the Research Ethics Committee of the Federal University of Rio Grande do Norte (No. 7.896.537).

The study population consisted of critically ill patients with high systemic complexity, including infectious diseases (e.g., HIV, tuberculosis, and meningitis) and metabolic disorders. A total of 13 patients admitted between January and December 2025 to the intensive care unit (ICU) of Hospital Giselda Trigueiro, a public referral hospital for infectious diseases in Northeastern Brazil, were included in the study. Inclusion criteria were: (1) clinical diagnosis of unilateral or bilateral angular cheilitis and (2) at least two dental evaluations during the ICU stay. Exclusion criteria included: (1) previous treatment for angular cheilitis before ICU admission; (2) presence of orotracheal tube positioning directly over the labial commissures, in order to avoid diagnostic confusion with traumatic lesions; (3) systemic or local contraindications to aPDT, including history of photosensitivity or known allergy to the photosensitizer agent; and (4) patients receiving palliative care with imminent risk of death. No age restrictions were applied, aiming to reflect the clinical reality of critically ill patients in the intensive care setting.

### Clinical evaluation and data collection

The initial clinical evaluation was performed at the patient’s bedside by a calibrated oral medicine specialist trained to identify the diagnostic criteria for angular cheilitis described below, using a flashlight to assist the oral examination. Sociodemographic and clinical data were collected from medical records and standardized forms developed for the study, including age, sex, comorbidities, reason for hospital admission, and systemic and/or topical antifungal therapy.

The physical examination of the oral and perioral regions was conducted to diagnosis of AC, based on the clinical criteria described by Chiriac et al. [6], considering the presence of erythematous areas, fissures, crusted or hyperkeratotic lesions, and angular ulcerations. Following the classification of, Chiriac et al. [6], four subtypes were defined based on lesion severity:

- Type I: Small fissures limited to the corner of the mouth, with slight involvement of the adjacent skin;
- Type II: Irregular borders, involving a more extensive area than Type I;
- Type III: Multiple fissures radiating from the corner of the mouth to the adjacent skin;
- Type IV: Absence of fissures, characterized by cutaneous erythema extending to the vermilion border of the lip.

**Fig. 1:**
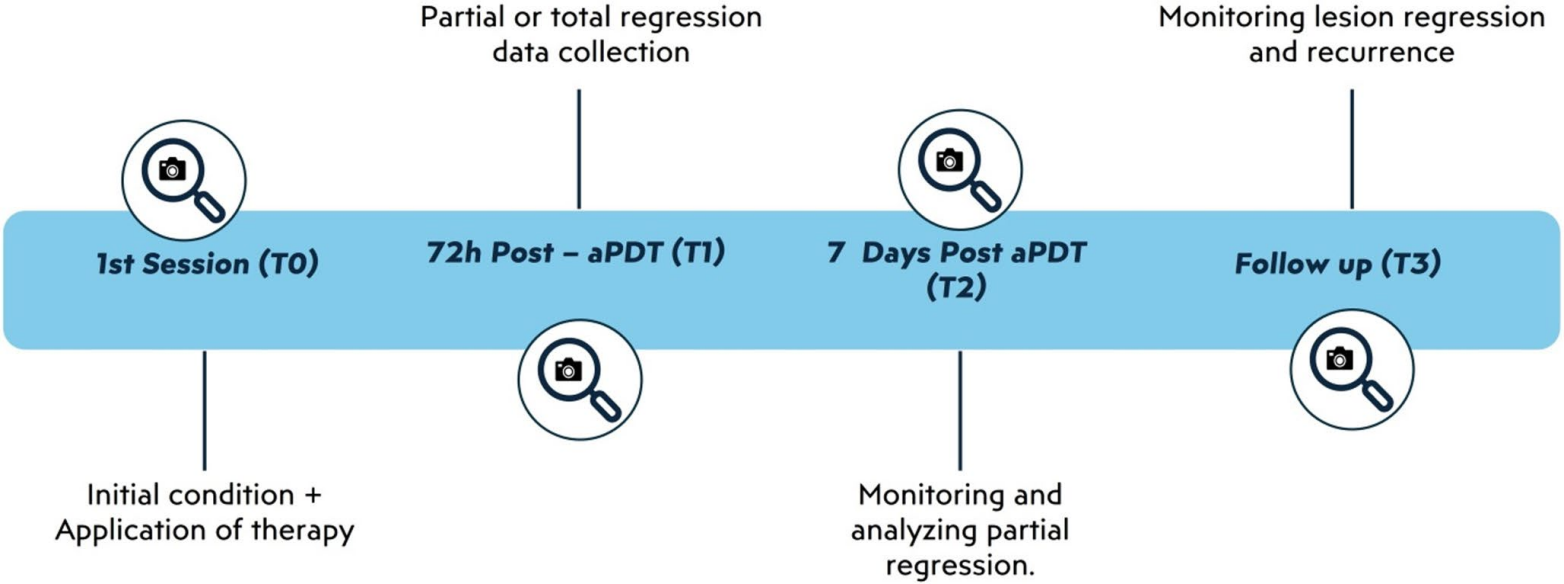
Timeline showing the sequential phases of regression monitoring

Treatment efficacy was assessed through clinical examination and classified as complete remission, partial remission, or absence of clinical resolution, according to tissue restoration and lesion persistence. Clinical progression was monitored through photographic documentation at T0 (baseline), T1 (72 h), T2 (7 days), and T3 (follow-up; 14 days after baseline), evaluating lesion regression and tissue re-epithelialization (Fig. 1).

### Antimicrobial photodynamic therapy protocol

The application protocol began with the preparation of the treatment area. The lesion was dried with sterile gauze, followed by the application of 0.1% methylene blue (MB) (ACS Científica, Sumaré, SP, Brazil). The photosensitizes was dispensed using 1 mL syringes onto cotton-tipped swabs and applied over the affected area and adjacent tissue. After a pre-irradiation period of 10 min, excess MB was removed with dry gauze.

**Fig. 2:**
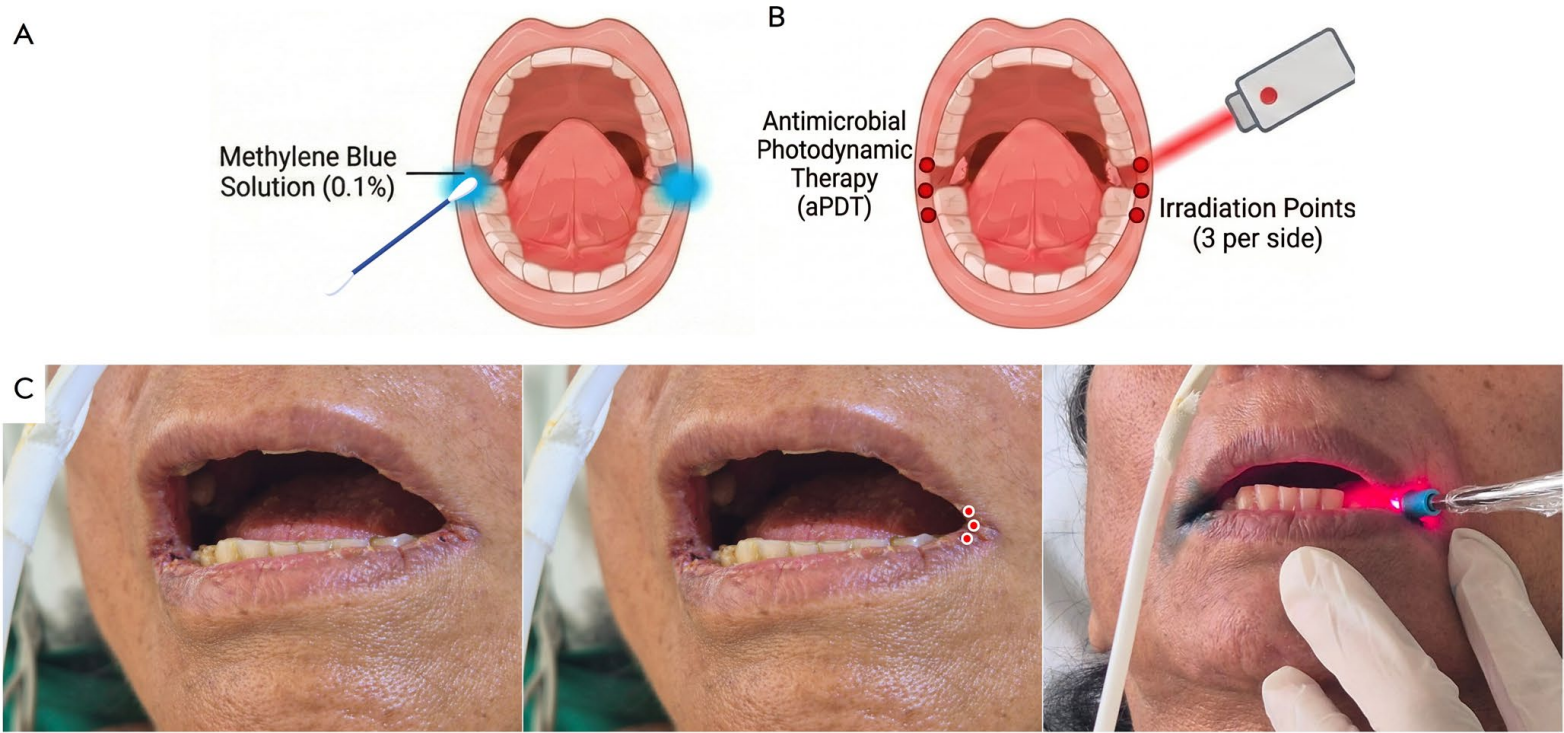
Photodynamic therapy application protocol. **a** 0.1% methylene blue application area; (**b**) Distribution of irradiation points (three points per affected commissure); (**c**) Clinical sequence (from left to right): initial lesion, schematic of planned irradiation points, and the irradiation procedure with the laser tip in contact with the lesion

Irradiation was performed using a low-level laser device (Therapy EC, DMC Equipamentos, São Carlos, SP, Brazil) in direct contact with the lesion, which was protected by a transparent physical barrier to prevent contamination. The protocol involved irradiating three points per affected labial commissure: one central point, one superior point, and one inferior point (Fig. 2). The exposure time was 40 s per point, delivering an energy of 4 J per point. Detailed technical specifications and additional parameters are presented in Table 1.

**Table 1:** Laser device specifications and irradiation parameters used for antimicrobial photodynamic therapy (aPDT)

Antimicrobial photodynamic therapy (aPDT) parameters	
Device	Therapy EC (DMC Equipamentos, SP, Brazil)
Type of emitters	Semiconductor diode (InGaAlP)
Wavelength	660 nm (red)
Output power	100 mW
Beam area	0.0028 cm^2^
Irradiation time	40 s
Energy	4 J
Number of points	3 points
Application technique	Application at one central point of the commissure, one superior point, and one inferior point to the affected area.
Total energy	12 J (per affected side)

## Results

A total of 13 patients were included in the study, with ages ranging from 26 to 85 years (mean age of 55 years). The majority of patients were female (*n* = 8; 61.5%). Regarding systemic involvement, the most frequent comorbidities were diabete mellitus (*n* = 5) and HIV infection (*n* = 4), occuring either alone or in association with other systemic conditions.

Reasons for hospital admission were heterogeneous, including systemic infections, respiratory decompensation, and cardiovascular complications. Most patients (*n* = 12; 92.3%) received intravenous fluconazole (150 mg/day for 5 days), and only one patient used topical nystatin solution for intraoral pseudomembranous candidiasis. Concomitant pseudomembranous candidiasis in other areas of the oral cavity was diagnosed in 10 patients (76.9%) (Table 2).

**Table 2:** Demographic and clinical profile of hospitalized patients

Case	Age/Sex	Reason for hospitalization	Comorbidities	Antifungal therapy	Concomitant oral candidiasis
1	77 F	Urinary tract infection	Hypertension and diabetes	Fluconazole (150 mg) + Nystatin 100,000 IU/mL	Yes (Pseudomembranous)
2	34 M	HIV disease (Infectious/Parasitic)	HIV	Fluconazole (150 mg)	Yes (Pseudomembranous)
3	26 F	HIV disease (Other)	HIV, asthma	Fluconazole (150 mg)	Yes (Pseudomembranous)
4	75 M	NR	NR	Fluconazole (150 mg)	Yes (Pseudomembranous)
5	53 M	Acute respiratory failure	Diabetes	Fluconazole (150 mg)	Yes (Pseudomembranous)
6	33 M	HIV disease (Kaposi’s sarcoma)	HIV	Fluconazole (150 mg)	Yes (Pseudomembranous)
7	49 F	Neurological disorder	Diabetes, history of ovarian cancer	Fluconazole (150 mg)	No
8	74 F	Diabetes mellitus (complications)	Hypertension and diabetes	Fluconazole (150 mg)	No
9	85 F	Hypertensive heart disease + CHF	NR	Fluconazole (150 mg)	Yes (Pseudomembranous)
10	62 F	Bacterial meningitis	NR	Fluconazole (150 mg)	Yes (Pseudomembranous)
11	60 F	Seizures	Diabetes	Fluconazole (150 mg)	Yes (Pseudomembranous)
12	59 F	HIV disease (Other)	HIV, Pre-diabetes, Hypertension	Nystatin 100,000 IU/mL	No
13	28 M	Tuberculosis	NR	Fluconazole (150 mg)	Yes (Pseudomembranous)

*Abbreviations*: *F* Female, *M* Male, *HIV* Human immunodeficiency virus, *CA* Cancer, *CHF* Congestive heart failure, *mg* Milligrams, *mL* Milliliters, *NR* Not reported

Regarding the clinical characteristics of AC, unilateral involvement was observed in 8 cases (61.5%). All patients exhibited classic signs of inflammation, including fissures or crusted areas at the labial commissures. In terms of severity, Types I and III were the most frequent, each accounting for 5 cases (38.4%), followed by Type II with 3 cases (23.0%). No Type IV lesions were observed.

At T1, 10 patients had available records for analysis. Four cases (cases 2, 4, 5, and 8; 40%) showed complete clinical regression, while 6 (60%) exhibited partial regression. By T2, complete resolution was maintained in 4 patients, and 4 additional patients progressed from partial regression or missing data to complete regression, totaling 8 patients (61.5%) with complete resolution. One case (case 7; 7.7%) remained with partial regression, and 3 cases (cases 6, 10, and 11; 30.8%) were lost to follow-up (Table 3).

**Table 3:** Characteristics of angular cheilitis and response to therapy

Case	Location of angular cheilitis (T0)	Severity classification	T1 evaluation (72 h post-aPDT)	T2 evaluation (7 days)	T3 evaluation (Follow-up)
1	Unilateral	Type II	Not recorded	Complete regression	Late success
2	Unilateral	Type II	Complete regression	Maintained (healed)	Immediate success
3	Unilateral	Type III	NR	Complete regression	Late success
4	Bilateral	Type I	Complete regression	Maintained (healed)	Immediate success
5	Unilateral	Type III	Complete regression	Maintained (healed)	Immediate success
6	Unilateral	Type I	Partial regression	Lost to follow-up	Partial improvement
7	Unilateral	Type III	Partial regression	Partial regression*	Partial improvement (healed at 15 days)
8	Bilateral	Type I	Complete regression	Maintained (healed)	Immediate success
9	Unilateral	Type I	Partial regression	Complete regression	Late success
10	Unilateral	Type III	Partial regression	Lost to follow-up	Partial improvement
11	Bilateral	Type III	Partial regression	Lost to follow-up	Partial improvement
12	Bilateral	Type I	NR	Complete regression	Late success
13	Bilateral	Type II	Partial regression**	NR	NR

*Abbreviations: aPDT *Antimicrobial photodynamic therapy, *AC* Angular cheilitis, *T0* Baseline session, *T1* Reassessment after 72 hours, *T2* 7 days, *T3* 14 days, *NR* Not reported

*The patient presented complete regression only after 15 days

**The patient presented unilateral complete regression and unilateral partial regression

**Fig. 3:**
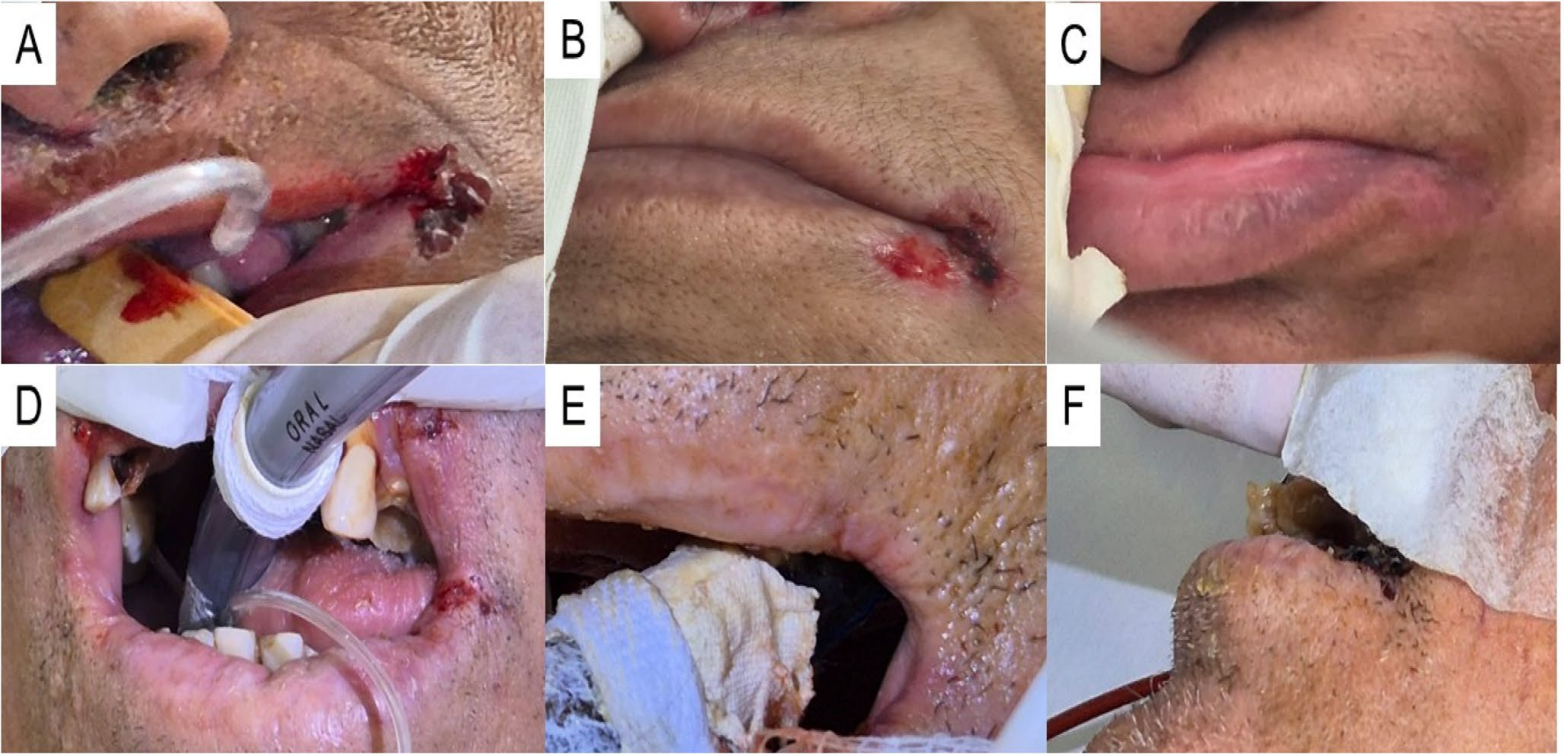
Clinical evolution of angular cheilitis following antimicrobial photodynamic therapy (aPDT) in two patients. Case 3 (**a**–**c**): (**a**) Baseline (T0) showing the initial lesion at the labial commissure, with marked erythema, fissuring, and ulceration; (**b**) 72-hour follow-up (T1) demonstrating a significant reduction in inflammatory signs and early tissue repair; (**c**) Follow-up (T3) showing complete re-epithelialization with no signs of recurrence. Case 1 (**d**–**f**): (**d**) Frontal view showing a lesion at the left labial commissure prior to the first aPDT session; (**e**) 72-hour follow-up demonstrating complete regression; (**f**) Lateral view at 7-day follow-up (T2) showing no signs of recurrence

At T3, clinical regression was confirmed in 8 patients (61.5%) (Fig. 3). Partial improvement was observed in 4 cases (30.8%), one of which (case 7) progressed to complete resolution at a later time point (day 15) (Table 3). Case 13 exhibited an asymmetric response (complete regression on one side and partial regression on the other) prior to the interruption of follow-up. No adverse effects were reported during or after the aPDT sessions.

## Discussion

To the best of our knowledge, this is the first case series to report the use of antimicrobial photodynamic therapy (aPDT) for the management of AC in critically ill patients. This study evaluated the efficacy of an aPDT protocol using 0.1% methylene blue as a photosensitizer in a cohort of 13 patients admitted to an intensive care unit at a reference infectious disease hospital in Northeastern Brazil. The findings demonstrated that aPDT, whether as monotherapy or as an adjunct to antifungal treatment, leads to favorable clinical outcomes without adverse effects. Complete clinical regression was observed within the first 72 h in 40% of patients, increasing to 61.5% at subsequent follow-ups (T2 and T3). The partial improvement in an additional 30.8% of cases further supports the potential of aPDT to accelerate infectious lesions resolution in critically ill patients, highlighting its potential as an effective and feasible therapeutic approach in the hospital setting.

Candidiasis is frequently described as a “disease of the diseased,” as its mucocutaneous manifestations are prevalent among immunocompromised individuals [2, 9]. These conditions reflect underlying systemic disorders, such as HIV/AIDS infection, uncontrolled diabetes mellitus, nutritional deficiencies, anemia, and hematological malignancies [3, 9]. According to Ferreira et al. [3], the clinical profile of patients in intensive care units (ICUs), including compromised clinical status, exposure to multiple invasive procedures, and inadequate oral hygiene, increases susceptibility to the development of opportunistic yeasts of the genus *Candida*. This opportunistic pathogen may lead to invasive candidiasis and candidemia, resulting in prolonged hospitalization and increased healthcare costs.

Thus, all patients included in this study exhibited some degree of immunosuppression, either related to the underlying condition leading to ICU admission or to the pharmacological treatments administered. Complications of diabetes mellitus (*n* = 5) and HIV infection (*n* = 4) were the most frequent comorbidities, reinforcing the relevance of AC as an infectious condition in critically ill patients.

Given the clinical importance of managing opportunistic oral infections and the rising resistance to conventional therapies, aPDT has emerged as a promising therapeutic alternative [10]. The persistence of lesions at baseline (T0), despite 92.3% of patients receiving systemic fluconazole, suggests that pharmacological therapy alone may be insufficient for managing these lesions in the ICU setting.

This therapy is based on the generation of reactive oxygen species (ROS), which induce damage to essential microbial structures [8]. The process involves the application of a photosensitizing agent to the infected area, followed by irradiation with a light source at a specific wavelength to activate the compound [8, 11]. Unlike systemic antifungal agents used by most patients in this study, ROS exert a multi-target effect on pathogenic fungi, damaging the cell membrane, proteins, and nucleic acids, thereby minimizing the risk of resistance development [8]. This mechanism may explain the favorable clinical response observed throughout the follow-up period in the present study.

Analysis of lesion severity, based on the criteria proposed by Chiriac et al. [6], revealed that clinical complexity directly influences therapeutic response time. While Type I and II lesions showed a tendency toward faster regression, patients with Type III lesions required additional aPDT applications and exhibited a longer healing time. These findings suggest that, in critically ill patients with comorbidities such as HIV infection and diabetes mellitus, aPDT may act not only by reducing the microbial burden through the generation of reactive oxygen species (ROS), but also by promoting local tissue repair. This dual effect is likely related to enhanced local decontamination and improved tissue conditions, which facilitate re-epithelialization and accelerate the healing process [2, 8].

In the present study, methylene blue (MB) was selected as the photosensitizing agent due to its low cost, favorable safety profile, rapid onset of action, and well-established antimicrobial activity [8]. The effectiveness of MB has also been demonstrated by Fonseca et al. [12] in a study evaluating the treatment of oral candidiasis in patients with head and neck squamous cell carcinoma (HNSCC). Compared to curcumin, MB showed superior therapeutic outcomes, including a significant reduction in the number of affected anatomical sites. These findings reinforce the potential of MB as a photosensitizer of choice, consistent with the results of the this study, in which clinical resolution was observed in the most of cases.

The efficacy of MB as a photosensitizer is supported by the findings of Casu et al. [5], who reported comparable outcomes for antimicrobial photodynamic therapy using either MB or curcumin, with both protocols achieving an 80% remission rate in cases of refractory angular cheilitis. Although curcumin has emerged as a promising natural photosensitizer, its clinical application requires a more complex preparation process, including powder hydration, homogenization, and a resting period to achieve adequate consistency and bioavailability. As highlighted by Casu et al. [5], these additional steps may compromise protocol standardization and reproducibility across different clinical settings. In contrast, MB is a well-established synthetic photosensitizer with standardized photochemical properties, proven antimicrobial efficacy, widespread availability, and low cost. Therefore, the selection of MB in the present study was based not only on its demonstrated effectiveness but also on its greater practicality and feasibility for routine clinical use, particularly in high-complexity healthcare environments [8, 12].

Furthermore, the effectiveness of aPDT as an adjunctive strategy for angular cheilitis management is consistent with the findings of de Souto Medeiros et al. [13], who reported favorable outcomes using this therapeutic approach in the treatment of denture stomatitis. In that clinical trial, the authors demonstrated a significant and immediate reduction in the microbial load of *Candida albicans and Staphylococcus spp*. following the first aPDT session, also using 0.1% methylene blue as the photosensitizing agent. These findings support our results, in which 40% of patients exhibited immediate complete regression. Together, these data highlight the potential of aPDT to rapidly and effectively disrupt complex biofilms, particularly in critically ill patients where conventional therapy alone may be insufficient.

Based on the literature and the findings in this study, the management of AC and oral candidiasis should not rely solely on topical therapies. Immunocompromised patients or those at risk of disseminated candidiasis, particularly critically ill individuals may benefit from the concomitant use of systemic antifungal agents [14]. A meta-analysis by Lin et al. [15] demonstrated that aPDT and conventional antifungal treatment show comparable efficacy in overall clinical response, with no significant differences in complete response rates. However, the combination of aPDT with antifungal therapy resulted in higher cure rates compared to antifungal treatment alone and was the most effective approach for reducing *Candida* burden. These findings support aPDT as a promising adjunctive therapy. Its implementation in hospital settings may not only enhance clinical resolution but also represent a safe strategy to reduce the recurrence and persistence of opportunistic infections in critically ill patients.

It is important to note that a particular feature of AC is its frequent association with bacterial infections, such as Staphylococcus aureus and *β-hemolytic streptococci*, likely due to the proximity to the skin [16]. Accordingly, the combined use of antibiotics and antifungal agents is conventionally recommended for the treatment of these lesions [17].

In this context, aPDT using methylene blue has also demonstrated bactericidal activity against *Staphylococcus aureus* and *Pseudomonas aeruginosa* in planktonic cultures, with reduced efficacy in biofilms. However, its bactericidal effect is enhanced when combined with gentamicin, particularly against *P. aeruginosa*, suggesting that, due to its antifungal and antibacterial potential activity, aPDT may act as an effective therapeutic approach for AC management [18].

This study has some important limitations that should be acknowledged. The small sample size and the clinical heterogeneity of critically ill patients may limit the generalizability of these findings. Specifically, the limited cohort size precluded a robust objective assessment of the therapy’s isolated effect in patients concurrently using systemic antifungal medications. Additionally, the dynamic clinical course inherent to the ICU setting presented challenges in ensuring consistent long-term follow-up, the 30.8% attrition rate reflects these intrinsic difficulties. Despite these constraints, the study provides valuable preliminary evidence of aPDT’s feasibility in high-complexity environments. Future research with larger cohorts is required to compare lesion-specific outcomes between unilateral and bilateral applications, further validating aPDT as a low-cost intervention to enhance wound healing in this vulnerable population.

Despite these limitations, aPDT using 0.1% methylene blue appears to be a safe and effective therapeutic approach for the management of angular cheilitis in the ICU setting. Its ability to promote both immediate microbial reduction and subsequent tissue re-epithelialization supports its use as an adjunct to conventional therapy, particularly in refractory cases or systemically compromised patients. In summary, these findings provide preliminary evidence for larger, controlled clinical trials, including comparative studies with other topical antifungal therapies, to further validate and standardize this approach within hospital-based oral care.

## Data Availability

No datasets were generated or analysed during the current study.

## Declarations

### Generative AI And AI-assisted technologies in the manuscript preparation process

In the course of preparing this manuscript, the authors employed ChatGPT (OpenAI, GPT-5.5 version) to assist in reviewing the English language and condensing the content. Following the use of this tool, the authors thoroughly reviewed and revised the material as necessary, and we assume full responsibility for the final content of the publication.
